# Does remission in systemic lupus erythematosus according to the 2021 DORIS definition match the treating rheumatologist’s judgement?

**DOI:** 10.1093/rheumatology/kead159

**Published:** 2023-04-11

**Authors:** Irene Altabás-González, Íñigo Rúa-Figueroa, Francisco Rubiño, Coral Mouriño, Íñigo Hernández-Rodriguez, Raúl Menor-Almagro, Esther Uriarte-Isacelaya, Eva Tomero, Tarek C Salman-Monte, Irene Carrión-Barberá, Maria Galindo-Izquierdo, M Esther Rodriguez-Almaraz, Luís S Inês, Norman Jiménez, José María Pego-Reigosa

**Affiliations:** Rheumatology Department, Complejo Hospitalario Universitario de Vigo, Vigo, Spain; IRIDIS (Investigation in Rheumatology and Immune-Mediated Diseases) Group, Galicia Sur Health Research Institute, Vigo, Spain; Rheumatology Department, Hospital Universitario de Gran Canaria Dr. Negrín, Las Palmas de Gran Canaria, Spain; Rheumatology Department, Hospital Universitario de Gran Canaria Dr. Negrín, Las Palmas de Gran Canaria, Spain; Rheumatology Department, Complejo Hospitalario Universitario de Vigo, Vigo, Spain; IRIDIS (Investigation in Rheumatology and Immune-Mediated Diseases) Group, Galicia Sur Health Research Institute, Vigo, Spain; Rheumatology Department, Complejo Hospitalario Universitario de Vigo, Vigo, Spain; Rheumatology Department, Hospital Universitario de Jerez de la Frontera, Cádiz, Spain; Rheumatology Department, Hospital Universitario de Donostia, San Sebastián, Spain; Rheumatology Department, Hospital Universitario de la Princesa, Madrid, Spain; Rheumatology Department, Hospital del Mar, Barcelona, Spain; Rheumatology Department, Hospital del Mar, Barcelona, Spain; Rheumatology Department, Hospital Universitario 12 de Octubre, Madrid, Spain; Rheumatology Department, Hospital Universitario 12 de Octubre, Madrid, Spain; Rheumatology Department, Centro Hospitalar e Universitario de Coimbra, Coimbra, Portugal; IRIDIS (Investigation in Rheumatology and Immune-Mediated Diseases) Group, Galicia Sur Health Research Institute, Vigo, Spain; Rheumatology Department, Complejo Hospitalario Universitario de Vigo, Vigo, Spain; IRIDIS (Investigation in Rheumatology and Immune-Mediated Diseases) Group, Galicia Sur Health Research Institute, Vigo, Spain

**Keywords:** SLE, remission, DORIS, target, Spanish, cohort, multicentre, disease activity

## Abstract

**Objectives:**

To assess agreement between the 2021 Definition Of Remission In SLE (DORIS) and physician-judged lupus activity.

**Methods:**

A cross-sectional analysis was conducted of data from a Spanish prospective multicentre study of SLE patients. We applied the 2021 DORIS criteria and assessed whether remission status based on this definition agreed with remission as per physician clinical judgement and reasons for disagreement between them.

**Results:**

Out of 508 patients [92% women; mean age (s.d.): 50.4 years (13.7)] studied, 267 (54.4%) met the criteria for 2021 DORIS remission. Based on physicians’ judgement, 277 (55.9%) patients were classified as in remission or serologically active clinically quiescent (SACQ). The overall rate of agreement between these assessments was 81.2% (95% CI: 79.9, 82.9%) with a Cohen’s kappa of 0.62 (0.55–0.69). Overall, 46 (9.1%) patients were classified as in remission/SACQ by rheumatologists but did not meet the 2021 DORIS criteria for remission. The main reasons for discrepancies were a clinical SLE Disease Activity Index (cSLEDAI) score >0 in 39 patients, a Physician Global Assessment score >0.5 in five patients, and prednisone >5 mg/day in another five patients.

**Conclusions:**

The 2021 DORIS remission is an achievable target in clinical practice. There is substantial agreement between the DORIS definition and physician-judged remission. The discordance was mainly due to physicians classifying some patients with ongoing mild disease activity as in remission. Thus, the standardized DORIS definition should be used to define the target in a treat-to-target strategy for the management of SLE.

Rheumatology key messages

- The 2021 DORIS is an achievable goal in clinical practice.
- There is substantial overall agreement between 2021 DORIS remission and physician-judged remission.
- The 2021 DORIS provides an appropriate target for clinical practice.

## Introduction

New strategies in SLE management have emerged in the last few years. Nonetheless, there is a need to improve long-term outcomes in SLE patients. Rates of mortality and organ damage remain high, with ∼50% of patients developing organ damage within 5 years of diagnosis [1, 2], and a mortality rate five times higher than in the general population [3]. Further, disease course, treatment and organ damage have a major impact on the health-related quality of life of these patients. Accordingly, the most recent European Alliance of Associations for Rheumatology (EULAR) recommendations for the management of SLE state that the main goals of treatment in SLE patients should be to control activity and flares, prevent damage accrual, minimize the side effects of drugs, especially glucocorticoids, and improve the quality of life and survival of patients with lupus [4].

A treat-to-target (T2T) approach in SLE patients has demonstrated several benefits for short- and long-term outcomes like prevention of organ damage, reduction in hospitalization and flare rates, and improvement in health-related quality of life and pregnancy outcomes [5–13]. When treating SLE patients, the main target should be remission or, when not achievable, low disease activity. The Lupus Low Disease Activity State (LLDAS), proposed by the Asia-Pacific Lupus Collaboration (APLC) is well validated [14]. Various definitions of remission have been proposed in the literature [15–18]. Recently, an expert task force developed the 2021 Definition of Remission In SLE (DORIS) [19].

To our knowledge, it has yet to be established whether the expert-driven DORIS criteria are concordant with treating physicians’ judgement concerning whether their patient is in remission in real-life clinical practice. A similar study was published from a single academic centre [20] by Mucke *et al.* where they observed discordance regarding DORIS and the treating physician judgement with a greater number of patients considered in remission by their physicians. The main reason of discordance between both was the failure in the PGA criteria.

Given this, the aim of our study was to evaluate the rate of 2021 DORIS remission in a large multicentre cohort of SLE patients in a clinical setting and its agreement with the clinical status of remission as judged by treating physicians.

### Study design

This study was a cross-sectional analysis of the baseline data of a prospective multicentre study. Seven Spanish rheumatology departments in tertiary university hospitals, with physicians experienced in the management of SLE patients, participated in the study. Data collection started in December 2018 and is ongoing. Data have been collected once a year for three consecutive years using an electronic case report form. A specific protocol was created to collect data on around 250 variables per patient. To ensure data homogeneity and quality, every item in the protocol has a highly standardized definition, and to minimize information bias, researchers attended a pre-trial training course and had online access to guidelines on how to complete the protocol. Ethical approval for this study was obtained from the Ethics Committee of Galicia (CEImG). All patients gave written informed consent before inclusion.

### Patients

We included consecutive adult SLE patients in an outpatient clinical setting who met the revised 1997 ACR classification criteria or the 2012 SLICC classification criteria for SLE [21, 22]. Patients under 18 years of age were excluded. To avoid selection bias, patients were recruited evenly across Spain.

### Variables

At recruitment, data were collected on demographic characteristics, SLE criteria, SLE clinical variables, the subjective assessment of disease activity by rheumatologists and patients themselves, and treatment. Subsequently, data on most of these variables have been collected yearly.

Specifically, disease activity was measured using the SLEDAI-2K referring to the previous 30 days [23] and the 28 tender/swollen joint count. Further, we recorded laboratory results of the SLEDAI-2K, ESR and CRP within 30 days of the visit. Clinical manifestations of SLE activity not included in SLEDAI were also collected in the online report form. These were: transverse myelitis, Libman–Sacks endocarditis, myocarditis, alveolar haemorrhage, shrinking lung syndrome, pneumonitis, alveolitis, thrombotic thrombocytopenic purpura and haemolytic anaemia. For any other clinical manifestation attributed to SLE not listed above, all investigators were allowed to write it in the report form, so all clinical manifestations of activity not included in SLEDAI could be captured. Organ damage was assessed using the SLICC/ACR Damage Index (SDI) [24].

The experienced rheumatologists were asked to categorize patients into one of five different clinical states, based on their assessment and clinical judgement: (i) remission; (ii) serologically active clinically quiescent (SACQ); (iii) low disease activity; (iv) moderate disease activity; or (v) high disease activity. Patients SACQ were defined as patients with positive serology anti-DNAds or low complement levels, without any clinical manifestation of SLE [25]. Physician Global Assessment (PGA) scores were obtained by asking physicians to rate disease activity on a scale from 0 to 3.

Treatment variables included: current use and dose of antimalarials, glucocorticoids, immunosuppressive treatments and biologic agents. Lastly, disease activity, as measured by the SLEDAI-2K score, at all patient visits was compared with the previous score and classified by the patient’s rheumatologist as showing: (i) clinically meaningful improvement; (ii) no clinically meaningful change in disease activity; or (iii) clinically meaningful worsening.

### Definitions of the different activity states

Based on the DORIS framework, several different definitions of remission were applied: clinical remission, complete remission, clinical remission on treatment (clinical ROT or 2021 DORIS) and complete remission on treatment (complete ROT) [26]. Clinical remission was defined as a clinical SLE Disease Activity Index (cSLEDAI) score of zero (excluding serology) and Safety of Estrogens in Lupus Erythematosus National Assessment (SELENA)-SLEDAI Physician Global Assessment (PGA; scale 0–3) <0.5, and complete remission as a SLEDAI of zero and a PGA <0.5 with no treatment except for antimalarials. When defining clinical remission and complete remission on treatment, the aforementioned definitions are applied but certain treatments are allowed including a prednisone dose ≤5 mg/day and well-tolerated standard maintenance doses of immunosuppressive drugs and approved biologic agents and antimalarials. Recently, the DORIS Task Force has recommended a single definition of remission in SLE, which is equivalent to clinical ROT [19].

### Statistical analysis

Sample size calculation*.* We calculated the number of patients needed for different levels of agreement considering that 60% of SLE patients are typically estimated to be in remission or have low disease activity. We estimated that we needed a sample size of 96 patients to obtain a statistical power of 80% with a significance level of 0.05.

Only the first patient visit was considered for the current study. Achievement of the 2021 DORIS remission was assessed according to the predefined criteria. Results were expressed as means (s.d.s) for continuous variables, and as numbers of patients (percentages) for binary and categorical variables. The ratings of global disease activity by physicians were divided into two categories: remission/SACQ *vs* low/moderate/high activity. These categories were then compared with the definition of remission of interest, i.e. 2021 DORIS, in a two-by-two table, assessing the agreement between DORIS and physician-judged remission/SAQC, using percentage of agreement and Cohen’s kappa as measures of agreement. The percentage of agreement was calculated as the number of matching scores divided by the total number of scores. Cohen’s kappa coefficient (κ, the agreement between measures beyond what would be expected by chance) was calculated to evaluate the degree of concordance/reliability between the two measures, where κ values of <0, 0–0.20, 0.21–0.40, 0.41–0.60, 0.61–0.80 and 0.81–1.0 indicate no, slight, fair, moderate, substantial and perfect agreement, respectively [27]. Cases in which there was disagreement were analysed further to evaluate which of the DORIS criteria contributed most to the discrepancy. Also, clinical manifestations not included in SLEDAI were analysed in cases of disagreement.

The threshold for statistical significance was set at *P* < 0.05. All analyses were performed with Hornik and R Core Team (2022) ‘The R FAQ’ (https://CRAN.R-project.org/doc/FAQ/).

## Results

### Demographics and disease characteristics

A total of 508 patients (92% women) were recruited. The mean (s.d.) age at diagnosis was 40.7 years (21), and at enrolment, the mean (s.d.) age and disease duration were 50.4 years (13.7) and 10.8 years (9.9), respectively. Disease history was described using the 2012 SLICC criteria and considering whether manifestations had ever been present. The most common clinical criteria were: arthritis, in 69.9% of the patients; cutaneous rash, in 62.8%; and leukopoenia, in 43.1%. A total of 491 patients (96.3%) were ANA positive, 64.8% had high anti-double-stranded DNA levels and 60.2% had low complement levels, while 167 patients (31.1%) had a history of lupus nephritis. At the time of the baseline visit, the mean (s.d.) SLEDAI-2K score was 2.8 (3.3) and the mean (s.d.) SDI score was 0.96 (1.36). More detailed information about the demographic and clinical characteristics of the cohort is presented in Table 1.

**Table 1.** Patient demographic and disease characteristics^a^

	Number (%) or mean (s.d.)
	(*n* = 508 patients)
Female	460 (92%)
Age at diagnosis (years)	40.7 (21.0)
Disease duration at enrolment (years)	10.8 (9.9)
Age at enrolment (years)	50.4 (13.7)
2012 SLICC criteria (ever present)	
*Clinical criteria*	
Acute cutaneous lupus	262 (51.8%)
Chronic cutaneous lupus	57 (11.2%)
Oral or nasal ulcers	171 (33.7%)
Non-scaring alopecia	156 (30.7%)
Arthritis	355 (69.9%)
Serositis	96 (18.9%)
Renal disorder	158 (31.1%)
Neurologic disorder	32 (6.3%)
Hemolytic anaemia	29 (5.7%)
Leukopenia	219 (43.1%)
Thrombocytopenia	88 (17.3%)
*Immunological criteria*	
Antinuclear antibody	489 (96.3%)
Anti-dsDNA antibodies	329 (64.8%)
Anti-Sm antibodies	95 (18.7%)
Anti-phospholipid antibodies	163 (32.1%)
Low complement levels	306 (60.2%)
Other disease characteristics at enrolment	
Number of ACR criteria for SLE	5 (1.5)
Number of SLICC criteria for SLE	6.24 (2.2)
SLEDAI-2K score	2.8 (3.3)
SLICC/ACR-DI score	0.96 (1.4)
Damage present	253 (49.8%)
Clinical SLEDAI-2 K (not including complement or anti-dsDNA level)	1.6 (2.7)
Hypocomplementemia	152(29.9%)
Elevated anti-dsDNA	125 (24.6%)
Physician Global Assessment score	0.2 (0.49)

dsDNA: double-stranded DNA; SLEDAI: SLE disease activity index; SLICC: Systemic Lupus International Collaborating Clinics; SLICC/ACR-DI: SLICC/ACR Damage Index.

^a^Recorded at enrolment on an ‘ever present’ basis.

In total, 371 (74%) patients were on antimalarials; 199 (39%) were on glucocorticoids, at a mean (s.d.) daily prednisone dose of 2.5 mg (5 mg); and 219 (44%) were receiving conventional immunosuppressants and/or biologics, Table 2.

**Table 2.** Treatments at the time of the enrolment

Treatment	Number (%)	Mean dose (s.d.)
Antimalarial	371 (74%)	253.4 mg/day (92.67)
Prednisone	199 (39%)	2.5 mg/day (5)
Methotrexate	57 (11.4%)	12.74 mg/week (6.28)
Mycophenolate mofetil	64 (12.9%)	1314.7 mg/day (664.2)
Mycophenolic acid	37 (7.4%)	911.1 mg/day (488.8)
Belimumab	39 (7.7%)	N/A
Azathioprine	32 (6.4%)	79.69 mg/day (46.85)
Rituximab	12 (2.4%)	N/A
Cyclophosphamide	4 (0.78%)	N/A
Ciclosporin	1 (0.2%)	N/A
Any immunosuppressant	219 (44%)	

### Rates of remission

The 2021 DORIS remission was achieved in 267 (54.4%) patients. Regarding other states of remission, clinical remission was achieved in 133 (27.3%) patients and complete remission in 118 (24.4%) cases, while complete remission on treatment (ROT) was achieved in 218 (46.4%). Further, a total of 304 (62.7%) patients were in LLDAS.

### SLE disease activity as judged by the treating rheumatologist

A total of 277 (55.9%) patients were classified as in remission (206 patients, 41.6%) or SACQ (71 patients, 14.3%) by their rheumatologist. On the other hand, 218 (44.1%) patients were classified as having low, moderate or high disease activity (Table 3).

**Table 3.** Lupus disease activity as assessed by their physician

Lupus disease activity	Patients (*n* = 508)
	*n* (%)
Remission	206 (41.6%)
Serologically active clinically quiescent	71 (14.3%)
Low activity	153 (30.9%)
Moderate activity	55 (11.1%)
High activity	10 (2%)

### Agreement between physician-judged remission/SACQ and 2021 DORIS remission

**Figure 1.**
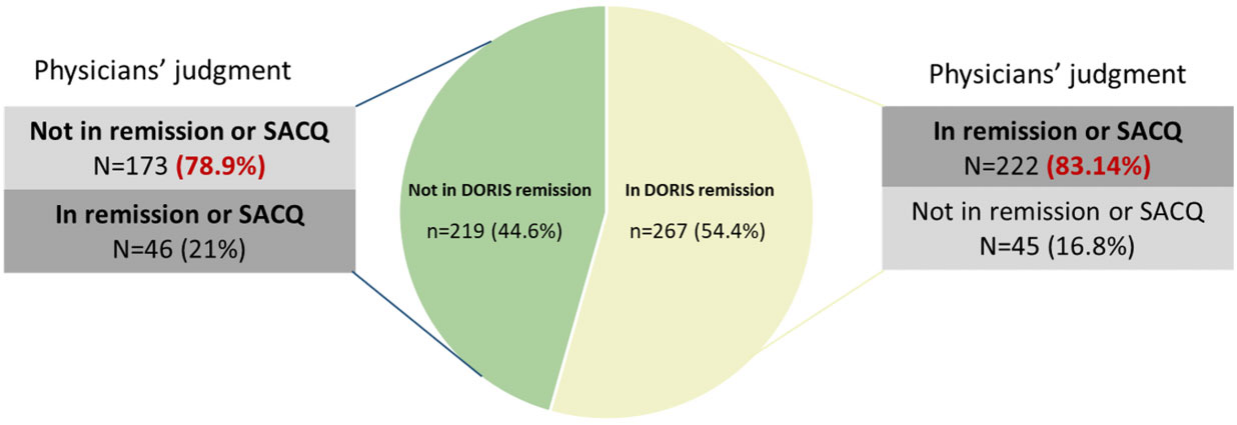
Comparison of DORIS remission and physician-judged remission/serologically active clinically quiescent (SACQ)

The overall agreement between physician-judged remission/SACQ and 2021 DORIS remission was 81.2% (95% CI: 79.9, 82.9%) with a Cohen’s kappa of 0.62 (0.55–0.69). Specifically, most patients in 2021 DORIS remission were also classified as in remission or SACQ by their rheumatologist (222 patients, 83.1%). On the other hand, among patients not in DORIS remission, rheumatologists classified 173 (78.9%) as not in remission and 46 (21.1%) as in remission or SACQ (Fig. 1). Looking for reasons to explain the disagreement in this last group, we observed that the criterion of cSLEDAI = 0 was not met in 39 out of the 46 patients (84.5%). For these patients (*n* = 39, i.e. those who were classified as in remission by their physician but who did not meet the 2021 DORIS criteria), we analysed the cSLEDAI in more detail. The median (IR) cSLEDAI was 3 (1–8). Leukopenia and alopecia were the most common manifestations, followed by oral ulcers. These data are reported in more detail in Table 4.

**Table 4.** Systemic Lupus Erythematosus Disease Activity (SLEDAI) criteria met by patients classified as in remission or SACQ by a rheumatologist but who did not meet 2021 DORIS remission criteria

SLEDAI criterion	Patients (*n* = 39)
	*n* (%)
Leukopenia	9 (19%)
Alopecia	9 (19%)
Oral ulcers	9 (19%)
Pyuria	6 (13%)
Hematuria	4 (8.6%)
Thrombocytopenia	3 (6.5%)
Rash	3 (6.5%)
Arthritis	3 (6.5%)
Proteinuria	2 (4.3%)
Pleurisy	1 (2.1%)

DORIS: definition of remission in SLE; SACQ: serologically active clinically quiescent; SLEDAI: SLE disease activity index.

The PGA <0.5 criterion was not met in only 5 (11.1%) cases, in which the mean (s.d.) PGA was 1.0 (0.008). Finally, the prednisone dose ≤5 mg criterion was not met in another 5 (11%) patients, who were taking a mean (s.d.) dose of 13.2 mg (6.418). Four of these patients had a cSLEDAI = 0 and one a cSLEDAI = 4, based on pyuria.

On the other hand, 45 out of 264 patients that did meet 2021 DORIS remission criteria were classified as not in a state of remission by their physician. Out of these 45 patients, three had activity manifestations not included in SLEDAI: cardiac (two) and gastrointestinal (one). On the other hand, 20 (44%) were receiving glucocorticoids, at a mean (s.d.) prednisone dose of 3.5 mg/day (1.39), and 35 (77.7%) were receiving immunosuppressants or biologics: 16 mycophenolate mofetil, six azathioprine, seven methotrexate, three belimumab and another three tacrolimus.

## Discussion

Management of SLE patients with a T2T strategy is necessary in clinical practice, as several studies have shown remission and low disease activity to be associated with improved long-term outcomes, namely, less damage accrual, fewer flares and hospitalizations, and better quality of life and pregnancy outcomes [5–12, 28]. In this study, we have evaluated the agreement in clinical practice between the physicians’ judgement of whether a patient is in remission and a standardized definition of remission proposed by the 2021 DORIS Task Force. We also analyse reasons for disagreement between the two assessments.

At baseline, about half of the patients met the criteria for 2021 DORIS remission. More stringent states of remission were more difficult to achieve, only about a quarter of patients being in clinical remission off treatment and in complete remission off treatment. Nonetheless, these results correspond to higher remission rates than in other SLE cohorts, which have reported rates of 11% to 45% [20, 29, 30]. Further, half of our patients were categorized as being in remission or SACQ by the treating physician. In >80% of cases, there was agreement between 2021 DORIS and physician-judged remission/SACQ. On the other hand, that still means that about 20% of patients not in remission according to the 2021 DORIS criteria were classified as being in remission/SACQ by their physician. The main reason for this disagreement was that the criterion of cSLEDAI = 0 was not met, in most cases, based on the presence of leukopenia, alopecia and oral ulcers. It seems that the vast majority of these patients had mild SLE manifestations that did not prevent the physician from considering them to be ‘in remission’. It may happen that, even fulfilling the definitions in the SLEDAI glossary, the expert considers these manifestations to be of little relevance from the clinical point of view, either because they are very mild manifestations or because they are stable analytical values close to normal (e.g. platelet count 98 000/mm^3^, borderline pyuria/hematuria levels), both without any clinical significance.

This is well illustrated by nine of these discordant cases having leukopenia (defined by SLEDAI as a white blood count of <3000/mm^3^), this not having major clinical or therapeutic implications and not being an impediment to rheumatologists considering the disease to be in remission.

On the other hand, only five patients were classified as in remission/SACQ by the rheumatologist but had a PGA >0.5, and hence, did not meet the 2021 DORIS remission criteria. The PGA in those patients was nearly 1. In a similar study, Mucke *et al.* [20] observed that the main reason of discordance was the PGA criterion; however, they used a different PGA scale (0–10) which was then converted to a 0–3 scale. Most of the patients (22 of 24) of the mentioned study who did not achieve DORIS remission due to the PGA >0.5 had a marginally elevated PGA of 0.6. These findings highlight the importance of using an adequate scale and the importance of an adequate instruction on its use. As we observed in a previous study in this cohort, the PGA seems to correlate well with the operational definitions of LLDAS and 2021 DORIS even though our cohort is multicentre and different rheumatologists were involved in patient assessment [31]. Nonetheless, although experts are trying to establish measures to be implemented homogeneously and universally when evaluating patients with SLE, both operational definitions of LLDAS and DORIS require a subjective evaluation of the physician (PGA). This could imply reliability issues. Further, as recommended by the authors of the Physician Global Assessment International Standardization Consensus in Systemic Lupus Erythematosus (PISCOS) study, the PGA should be scored by the same physician at all visits [32], a condition which may not be satisfied in clinical practice. This could lead to inconsistencies between different evaluators and a patient inappropriately not being classified as in remission only because they do not meet this subjective condition. Other definitions of low activity or remission that do not include this criterion [15, 16] might show greater agreement with the physician's judgement, and it would be interesting to explore this in a future study.

In the current study, only five patients (11%) failed DORIS remission due to a prednisone dose >5 mg/day. The mean prednisone dose of the five patients exceeded 10 mg/day, and for most of them, the cSLEDAI was 0. However, in another similar study [20], although the mean dose of prednisone was 3 mg/day, a high number of patients (28%, *n* = 37) failed DORIS remission due to the prednisone criterion.

Regarding those five patients, we suspect that they could be taking a higher dose of glucocorticoid for a reason other than SLE activity (e.g. one patient taking >5 mg of prednisone a day because of an exacerbation of her chronic obstructive pulmonary disease). Another explanation of the disagreement in these patients can be the fact that these patients’ disease becomes active when they reduce their prednisone dose to 5 mg/day so that a higher dose may be used as a preventive dose of flare.

Regarding the glucocorticoid criterion, we obtained interesting real-world data. Notably, 60% of patients were not taking any glucocorticoids and those who were tended to be on a low dose. These data demonstrate that most SLE patients can be effectively well managed without glucocorticoids or with no >5 mg/day of prednisone, which is in accordance with current EULAR management recommendations [4]. It might also be explained by physicians being more aware of the side effects of glucocorticoids in SLE patients and also wider use of other sparing therapies and biologics in SLE.

In our study, we observed substantial agreement between physician-judged remission or SACQ and a standardized definition of remission, something that we did not observe for LLDAS [31]. This suggests that physicians can appropriately classify patients who are in remission but not those in LLDAS. Discordances between LLDAS and low disease activity as judged by physicians were mainly observed in the criterion concerning new features of lupus (comparing current SLE activity with that at the previous assessment) and the SLEDAI score. Notably, the 2021 DORIS definition does not take into account the status of the patient at the previous visit. Regarding the disease activity index, this criterion differs between the two definitions (cSLEDAI being used for DORIS and SLEDAI for defining LLDAS), and therefore, any mild clinical SLEDAI manifestations might imply not meeting the LLDAS definition in patients with serological activity. Both for LLDAS and 2021 DORIS remission, it could be that physicians do not place much importance on the presence of certain clinical manifestations included in the SLEDAI, and this would explain why the SLEDAI criterion emerges as a relevant cause of discordance.

We analysed reasons for disagreement in the case of patients who met the 2021 DORIS remission criteria but were not classified as in remission or SACQ by physicians in more detail. The vast majority of these patients were on treatment with immunosuppressants and/or biologics, and hence, it may be that the physician does not consider them to be in remission because their low SLEDAI scores are achieved at the cost of more intensive treatment. This suggests that some physicians only consider a patient to be in remission when they do not require any treatment apart from antimalarials and low-dose glucocorticoids.

Some strengths of our study are: it is a well-designed multicentre cross-sectional study with a large sample of SLE patients included; all investigators were previously instructed and trained in the protocol; and all had access to an online report form so data were homogeneously collected.

Our study is limited by the cross-sectional nature of data from a single visit. In future longitudinal analysis of our cohort, we will assess whether these states of remission and LLDAS have been maintained over time and their relationship with long-term outcomes.

In conclusion, we found that 2021 DORIS remission is an achievable target in the management of SLE patients in clinical practice. There is a substantial agreement between physician-judged remission and the standardized DORIS definition. The main reason for the discrepancy in patients who do not achieve DORIS remission is the cSLEDAI score being >0, mainly due to ongoing mild activity. For applying a T2T strategy, it is better to use a standardized measure than one based on clinicians’ judgement. In particular, it would enable more consistent management in clinical care, recognizing that different physicians provide care (to the same patient over time and to different patients), and they may differ in what they consider remission, leading to a risk of advising erratic treatment changes. Applying a standardized measure over longitudinal follow-up can be expected to ensure more consistent disease assessments and treatment decisions, and this would benefit patients.

## Data Availability

The data underlying this article will be shared on reasonable request to the corresponding author.
